# Identification of foundational non-clinical attributes necessary for successful transition to residency: a modified Delphi study with experienced medical educators

**DOI:** 10.1186/s12909-018-1247-6

**Published:** 2018-06-22

**Authors:** Stephen J. Wolf, Tai M. Lockspeiser, Jennifer Gong, Gretchen Guiton

**Affiliations:** 1 Department of Emergency Medicine, University of Virginia School of Medicine, PO Box 800699, Charlottesville, VA 22908 USA; 2 Department of Pediatrics, University of Colorado School of Medicine, Aurora, CO USA; 3 Department of Family Medicine, University of Colorado School of Medicine, Aurora, CO USA; 4 Department of Internal Medicine, University of Colorado School of Medicine, Aurora, CO USA; 5 Office of Undergraduate Medical Education, University of Colorado School of Medicine, Aurora, CO USA

**Keywords:** Undergraduate medical education, Residency transition, Non-clinical attributes, Graduate medical education

## Abstract

**Background:**

We aimed to identify foundational non-clinical attributes expected of medical school graduates to be successful in residency.

**Methods:**

We conducted a three-round modified Delphi study with snowball sampling of experienced medical educators. In Round 1, respondents rated 28 attributes identified from a literature search. Additional attributes were proposed through invited comments. In Round 2, respondents expressed their agreement with advanced attribute definitions and examples. Consensus on final definitions and examples was obtained in Round 3.

**Results:**

Sixty-four percent (105/163) of invited educators participated in Round 1. There was broad representation of educational focus (undergraduate, graduate, and continuing medical education) and field of practice (primary care, sub-specialty, medical, and surgical). Thirteen attributes were advanced to Round 2. Ninety-seven of 105 (92%) respondents participated in Round 2, with greater than 92% agreement for all attributes. Three pairs were consolidated. In Round 3, 88% (85/97) of educators expressed greater than 92% agreement about definitions and representative examples. The final 10 foundational attributes are: communication skills, critical thinking, emotional intelligence, ethical behavior, intellectual curiosity, organizational skills, resilience, self-improvement, teamwork, and vocational commitment.

**Conclusion:**

Through a consensus-building process of medical educators, we identified and defined 10 foundational non-clinical attributes for a medical student’s successful transition to residency.

## Background

Calls for reform in medical education emphasize the need for more standardized learning outcomes that foster greater integration across the educational continuum [1, 2]. Achieving this vision of integration between undergraduate and graduate medical education requires a thorough understanding of the attributes and competencies expected of medical school graduates. Clearly defined outcomes can then be used to inform a competency-based approach to curriculum development, teaching, and assessment throughout the entirety of medical school [2]. In particular, accepted attributes and competencies for the successful transition from medical school to residency will better guide curriculum design for the final, and often underutilized, year of medical school which could potentially transform it into a lynchpin for successful integration of the medical education continuum [3–5].

National efforts have made great strides towards defining the competencies needed for students’ successful transition to residency. The American Association of Medical Colleges has engaged in two efforts to more thoroughly define competencies appropriate for medical students – the Physician Competency Reference Set (PCRS) and the Core Entrustable Professional Activities for Entering Residency (core EPAs) [6, 7]. While both efforts have the goal of standardizing learning outcomes in medical school, the core EPAs have the added goal of fostering successful student preparation for residency by clearly delineating activities that all graduating medical students should be expected to perform on day one of residency without direct supervision [7]. Initiatives to implement the core EPAs are underway at numerous medical schools around the country as a means of trying to improve medical student preparation for residency [8, 9]. Importantly, both of these competency frameworks rely on foundational attributes, or habits of the mind, which are seen as essential to determining ultimate competence or entrustment. For instance, competencies have been framed as requiring attentiveness, critical curiosity, self-awareness and presence, while EPA’s require trustworthiness and self-awareness [10].

Studies across multiple specialties repeatedly support the notion that non-clinical attributes are critically important to successful performance in residency [11–18]. Examples of these behaviors include having a commitment to learning, being conscientious, being curious, recognizing one’s limits, exhibiting professional behavior, and having strong interpersonal skills [11–14]. One study suggests that only 30% of attributes related to successful performance are those traditionally thought of as cognitive (i.e., knowledge and technical skill related) [12]. Another study identifies multiple non-clinical attributes as contributors of difficulty for new interns, including lack of self-reflection skills or poor organizational skills [18].

Unfortunately, many of these foundational non-clinical attributes are less well studied in medical education, often only indirectly addressed in clinical training, and subsumed into the broader PCRS and core EPAs. So, while these current efforts are necessary to ensure a student’s successful transition to residency, they may insufficiently emphasize non-clinical habits of mind which have also been shown to confer success in residency [11–18]. This success clearly depends on competencies and behaviors that extend beyond the cognitive elements of medical knowledge and patient care [6, 14].

In this study, we set out to systematically identify and define the foundational non-clinical attributes expected of medical school graduates necessary for success in residency training. In doing so, we hope to inform individualized student learning programs, curricular design, and final-year scheduling. Using a modified Delphi method of expert consensus, a snowball sample of experienced educators participated in defining the foundational, non-clinical attributes expected of all medical school graduates.

## Methods

### Study design

Three rounds of a modified Delphi procedure, with snowball sampling of faculty with medical education expertise, were used to identify and define non-clinical attributes expected of medical school graduates to be successful in residency training. The Delphi technique is a type of exploratory analysis in which multiple rounds of surveys are used to obtain a reliable consensus from a group of experts [19, 20]. A characteristic hallmark of this method is the use of the participants’ responses from previous rounds as the basis for subsequent rounds. Only those who participate in the previous round are invited to participate in a subsequent round. The success of a Delphi process is dependent on continued participation throughout multiple rounds. Unlike a standard Delphi process, our methodology was modified in that the researchers provided an initial list of attributes for the participants and generated definitions for subsequent rounds for consideration by the Delphi participants. All materials considered by the Delphi participants were developed by the research team through an iterative discussion process based on the participants’ responses from previous rounds. This study was approved by the Colorado Multiple Institutional Review Board.

### Subjects and sampling

Participants for this study were drawn from a systematic cross specialty sample of experienced educators at the University of Colorado. All invited participants had a significant educational role in undergraduate, graduate, or continuing medical education as a core educator, mentor, or program leader. We sought to include a diverse group of faculty in terms of the training level of their educational focus (undergraduate, graduate, and continuing medical education) and their field of practice (primary care, sub-specialty, medical and surgical). See Table 1 for the characteristics of study participants. The research team monitored participation to assure the inclusion of varying perspectives across each round of the modified Delphi process.

**Table 1:** Characteristics of study participants

	Percentage of Respondents		
	Round 1 *N* = 105 (%)	Round 2 *N* = 97 (%)	Round 3 *N* = 85 (%)
Female	51 (49)	53 (55)	51 (60)
Primary Care	50 (48)	44 (45)	42 (49)
Subspecialty Care	56 (52)	53 (55)	43 (51)
Medical-based Specialty	72 (69)	67 (69)	56 (66)
Surgical-based Specialty	33 (31)	30 (31)	29 (34)
Self-reported Educational Focus*			
UME	45 (43)	43 (44)	41 (48)
GME	71 (68)	66 (68)	65 (76)
CME	46 (44)	43 (44)	42 (49)

*UME* Undergraduate Medical Education, *GME* Graduate Medical Education, *CME* Continuing Medical Education

*Respondents may have selected more than one educational focus area

The research team generated an initial list of 45 faculty felt to have expertise in undergraduate, graduate, and continuing medical education. Snowball sampling then allowed the initial participants to identify additional faculty who they thought could contribute to this process. These additional participants were then added to the initial list and invited to participate in the process. No incentives were provided for participation in the study.

### Modified Delphi process

**Fig. 1:**
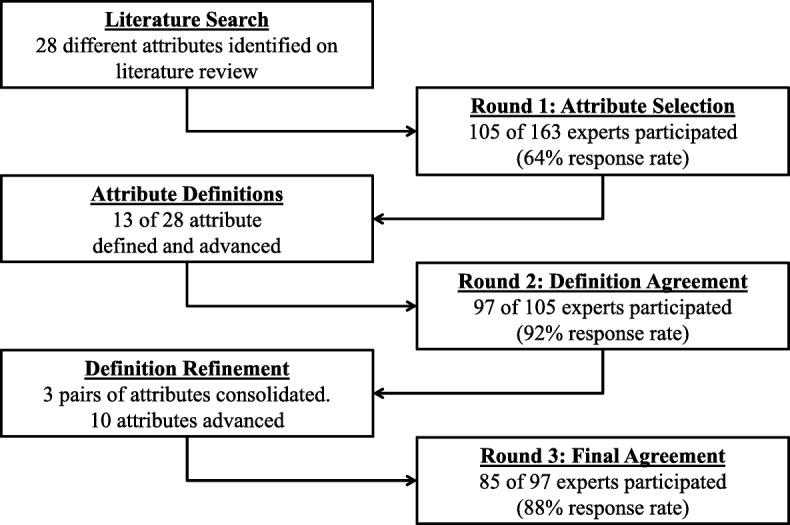
Overall steps in modified Delphi process

An extensive literature search was performed in 2011 to identify potential undergraduate medical education non-clinical attributes necessary for success in residency. Relevant search terms included: fourth year, undergraduate medical education, competency-based education, professional competence, and graduate medical education. Abstracts were reviewed and relevant articles were read entirely. Reviewing the bibliographies of the initial articles identified additional relevant articles to include. This search, and accompanying discussions among the researchers, was used to create an initial list of non-clinical attributes for use in the first round of the modified Delphi process. In 2012, an online survey was then created for each round of the modified Delphi process. Figure 1 contains the steps of the modified Delphi process.

The purpose of the first round was to identify the non-clinical attributes necessary for success in residency. We asked participants to react to the research team’s generated list of attributes from our review of the literature, rather than generate their own list. An open-ended question, however, allowed the respondents to suggest other attributes not included in the original list. Respondents rated the importance of each of the potential attributes on a six-point Likert scale from “not at all important” to “extremely important.” See Table 2 for list of initial attributes. In addition, respondents were asked to select the five most essential and the five least essential attributes for success in residency training.

**Table 2:** Means and frequencies of responses regarding each attribute following the three rounds of the modified Delphi process

Round 1 (n=105)				Round 2 (n=97)				Round 3 (n=85)		
Attribute	Mean Overall Rating (1-6)	Most Important (%)	Least Important (%)	Agree (%)	Agree with Modifications (%)	Disagree (%)	Recommend combine (%)	Agree (%)	Disagree (%)	Final Attribute Name
Communication Skills	5.5	58	2	80	19	1	6	93	7	Communication Skills
Teamwork	5.4	39	2	78	22	0	2	99	1	Teamwork
Resilience	5.4	29	5	75	23	2	3	95	5	Resilience
Critical Thinking	5.4	38	1	87	9	4	11	99	1	Critical Thinking
Clinical Reasoning	5.2	41	5	75	23	2	8			Critical Thinking
Vocational Commitment	5.2	13	12	85	11	4	4	94	6	Vocational Commitment
Prioritization of Tasks	5.2	26	9	70	23	7	27	96	4	Organizational Skills
Empathy	5.1	22	6	81	16	3	11			Emotional Intelligence
Emotional Intelligence	5.1	21	9	83	16	2	8	95	5	Emotional Intelligence
Time Management	5.1	24	2	81	18	1	25			Organizational Skills
Ethical Decision Making	5.1	20	8	92	9	0	3	99	1	Ethical Behavior
Self-Improvement	5.1	17	1	82	18	0	6	97	3	Self-Improvement
Intellectual Curiosity	5.0	21	10	85	13	2	6	97	3	Intellectual Curiosity
*Skills in Seeking Information*	*4.9*	*11*	*15*							
*Multitasking*	*4.8*	*13*	*31*							
*Learning Beliefs Motivation*	*4.8*	*5*	*31*							
*Mindful/Deliberate Practice*	*4.8*	*6*	*28*							
*Focus on QSPC*	*4.7*	*15*	*23*							
*Self-Reflection*	*4.7*	*7*	*16*							
*Attention to Learning Opportunities*	*4.6*	*7*	*27*							
*Professional Citizenship*	*4.6*	*8*	*29*							
*Wellness*	*4.6*	*6*	*25*							
*Self-Efficacy*	*4.5*	*5*	*19*							
*Reflective Practice*	*4.5*	*6*	*21*							
*Self-Concept*	*4.4*	*6*	*24*							
*Cultural Competence*	*4.2*	*1*	*35*							
*EBM Skills*	*4.1*	*1*	*45*							
*Leadership*	*3.7*	*2*	*59*							

*Italicized attributes were NOT advanced to Round 2; *QSPC* Quality and Safety in Patient Care, *EBM* Evidence-Based Medicine

Criteria for advancement of attributes to Round 2 were developed, based on iterative discussions among the researchers, with the goal of accurately reflecting the priorities of the Delphi participants. Ratings for each attribute and comments were reviewed. Respondent characteristics were reviewed to assure that sampling was adequately diverse prior to ending Round 1. After discussion, researchers agreed to advance attributes to Round 2 based on a mean importance score of greater than 5, with consideration of percent of respondents naming an attribute as the most and least important.

Definitions and representative examples for all the attributes included in Round 2 were developed through an iterative process in which all the researchers reviewed comments from the participants in the first round and the articles from the literature search. The purpose of the second round was to achieve agreement among proposed definitions and examples. Respondents from the first round were asked to agree, agree with modifications, or disagree with the definition of each attribute. Respondents were also asked if any attributes should be subdivided into multiple other attributes or if multiple attributes should be combined into one. Informed with these responses and comments, the research team engaged in iterative discussions in order to achieve consensus regarding which attributes were to be subdivided or combined before advancing to Round 3. Additionally, attribute definitions and representative examples were refined for clarity.

The purpose of Round 3 was to achieve final agreement on the list of attributes, their definitions, and representative examples. All attributes, either in their original or combined forms, were advanced in this round. Respondents from the second round were asked to indicate their level of agreement with the final attributes, definitions, and representative examples.

## Results

The systematic literature search yielded 48 relevant articles that were reviewed by the researchers and an annotated bibliography was created. Based on these articles, we compiled a list of 28 non-clinical attributes relevant to undergraduate and graduate medical education.

After snowball sampling, 163 experienced educators were invited to participate. One hundred and five (105) of the 163 faculty participated (64% response rate) in the first round. The overall mean rating of importance of all attributes was 4.84 out of 6 (standard deviation = 0.89). Based on discussion of the data, 13 attributes were advanced to the second round (see Table 2).

In Round 2, 97 of 105 participants from Round 1 responded (92% response rate). Between 70 and 90% of respondents agreed with the definitions provided and representative examples of each attribute (see Table 2). Based on comments from the respondents about overlaps, three sets of attributes were consolidated (clinical reasoning into critical thinking; empathy into emotional intelligence; time management and prioritization of tasks into organizational skills)**.** The definitions and examples of the remaining attributes were refined, incorporating perspectives from the respondents’ comments during this round.

In Round 3, 85 of 97 experienced educators responded (88% response rate), and there was greater than 92% agreement about the definition and representative examples for all attributes (see Table 2). Based on the results of this final round, we defined 10 non-clinical attributes that are expected of medical school graduates for success in residency training: communication skills, critical thinking, emotional intelligence, ethical behavior, intellectual curiosity, organizational skills, resilience, self-improvement, teamwork, and vocational commitment (see Table 3 for attribute definitions with representative examples).

**Table 3:** Definition and examples of non-clinical attributes expected of graduating students at the University of Colorado, School of Medicine

Attribute	Definition	Representative Examples
Communication Skills	Collect and convey information in order to create and sustain relationships with others in a contextually appropriate manner.	i. Employ appropriate non-verbal communication/body language
		ii. Develop and use relationship building skills
		iii. Give effective oral presentations
		iv. Create effective written communication
		v. Demonstrate active listening
Critical Thinking	Develop and use a process of thinking that incorporates information, seeks factual and contextual validity, and uses this knowledge to determine a best course of action.	i. Employ effective, developmentally appropriate clinical reasoning (i.e., generating an appropriate initial list of differential diagnoses based on the patient’s presenting signs and symptoms; modifying the initial list to reflect the information gleaned from the history, physical examination, and standard laboratory tests; identifying appropriate additional tests or historical information needed to reach the diagnosis; and determining when the information obtained is sufficient to make a provisional or a final diagnosis and to justify beginning therapy.)
		ii. Generate and convey a systematic approach to solving a problem
		iii. Raise questions regarding assumptions made by self or others
		iv. Consider conclusions in relation to context
		v. Justify and assess a range of possible answers
Emotional Intelligence	Accurately recognize and understand one’s own emotions and those of others, using this information to guide future behavior.	i. Recognize and monitor the importance of non-verbal communication
		ii. Understand and control reactions to the behaviors and emotions of others
		iii. Recognize and monitor the impact of emotions on decision making
		iv. Display empathy by understanding and appreciating the feelings, emotions, and perspectives of others
		v. Maintain healthy emotional boundaries
		vi. Strive to understand differing perspectives
Ethical Behavior	Demonstrate a commitment to just principles pertaining to patient autonomy and professional responsibilities.	i. Display honesty and truthfulness
		ii. Accept and acknowledge personal error
		iii. Document and report clinical information truthfully
		iv. Uphold confidentiality of patient information
		v. Use principles of informed consent
Intellectual Curiosity	Possess an intrinsic motivation to investigate, understand and apply knowledge.	i. Have a desire to know details, context and the broader picture
		ii. Have an open mind to alternatives
		iii. Incorporate and synthesize new and existing knowledge
		iv. Take advantage of learning opportunities
Organizational Skills	Prioritize and accomplish tasks in an efficient and effective manner.	i. Exhibit conscientious attention to details in all tasks
		ii. Demonstrate the ability to focus on the task at hand
		iii. Demonstrate adaptability and flexibility to complete a process or set of tasks
		iv. Appropriately prioritize and complete assigned work within time constraints
Resilience	Moderate negative effects of stress, promote adaptation, and successfully cope with change or adversity	i. Moderate Adapt to settings of increased workload or obligations
		ii. Adopt a growth mindset (i.e., learning and moving forward from mistakes, criticism, or negative experiences)
Self-Improvement	Engage in cognitive, physical, or psycho-behavioral activities, with the goal of improving one’s own knowledge, skills, and attitudes.	i. Identify strengths, deficiencies, and limits
		ii. Set learning and improvement goals
		iii. Ask for help when needed
		iv. Commit to professional growth through life-long learning
Teamwork	Effectively and respectfully work with others to achieve a common goal.	i. Demonstrate appropriate flexibility and adaptability in a team setting
		ii. Recognize and coordinate one’s roles and responsibilities with those of other team members
Vocational Commitment	Strive to excel in all professional responsibilities, both as a physician and in one’s specialty.	i. Lead by example
		ii. Demonstrate a strong work ethic
		iii. Take initiative with needed actions
		iv. Put professional responsibilities above personal responsibilities when appropriate

## Discussion

Based on input from experienced educators in undergraduate, graduate, and continuing medical education, we identified and defined 10 foundational non-clinical attributes necessary for a medical student’s success in residency. Derived from individual educators and across various specialties, these attributes can be considered fundamental to working in almost all clinical settings a new resident might encounter. As such, these attributes could be used to further define and augment medical school curricula and better prepare medical students for residency training.

To date much literature has been published about the competencies and competency domains needed for the successful completion of medical school and graduate medical education [5–9, 21]. One recent article reviewed the literature on competency frameworks and proposed a common taxonomy of competencies for physicians that includes eight domains and 58 competencies in eight domains (i.e., the Physician Competency Reference Set) [6]. Within this taxonomy, non-clinical attributes and competencies are encompassed across multiple domains, emphasizing their importance. All but two of the non-clinical attributes identified in our study can be mapped within the PCRS taxonomy, albeit sometimes indirectly. For example, in our study, emotional intelligence emerged as an independent attribute, while it is subsumed by interpersonal and communication skills in the PCRS. Two of our attributes, vocational commitment and organizational skills, are not explicitly included in the PCRS. The differences between our results and the PCRS may be attributed to the explicit goal of our study to determine the non-clinical attributes for success in residency, rather than a comprehensive list of competencies. This resulted in a more granular list of specific attributes that can be fostered and developed during the fourth year of medical school to support the transition to residency. Regardless, this similarity in results provides confirming evidence for content validity and further supports the findings of our study.

The Core Entrustable Professional Activities for Entering Residency (Core EPAs) is another competency framework related to ours [7]. This framework identifies 13 activities central to all residents that medical students should be able to complete prior to the start of residency and that are designed to holistically assess medical student performance in preparation for residency. Importantly, the core EPAs do not represent all that is expected from medical school graduates.

Our attributes were derived in a way that emphasizes the non-clinical attributes rather than the clinical activities necessary for residency. In addition, there is similarity between the findings of our study and the Accreditation Council for Graduate Medical Education (ACGME) Milestones for residency training [22–24]. For example, the ten attributes we identified are included in the 21 pediatric milestones to be reported every 6 months to the ACGME [22]. Similar to our emphasis on non-clinical attributes, of the 21 pediatric milestones, only 6 are related to patient care or medical knowledge, while the other 15 reflect the multitude of non-clinical competencies necessary for a pediatrician [22]. As such, we believe our attributes can be used to supplement efforts to implement the core EPAs and Milestones for both teaching and assessment.

Ultimately, we believe our 10 non-clinical attributes should be emphasized throughout all four years of medical school, not simply during the fourth year. Competency-based medical education involves starting with the end in mind and defining the competencies expected of graduates which can then inform curriculum development [23]. The idea that these attributes should be addressed throughout medical school is supported by a recent study of the core personal competencies considered in medical school admissions processes [25]. The competencies identified in that study are very similar to our findings, which are also similar to the findings from the critical incident studies for residency education, further supporting the importance of this set of skills [11–18].

The attributes identified in our study can be used in future studies to examine prospectively how non-clinical attributes may impact performance in residency education. In addition, the perspectives of other stakeholders, such as students or residents, about the importance of these attributes would further enhance our findings. Finally, efforts to foster and develop these attributes in medical student prior to medical school graduation should be developed and studied.

### Limitations

There were several limitations to our study. First, it was a single institution study, only engaging faculty working within a single culture and single approach to the fourth year of medical school. Faculty at different institutions may prioritize other attributes expected of medical school graduates necessary for success in residency training. Second, we opted to start with a pre-defined list of attributes rather than ask an open-ended question for respondents to list attributes on their own. This modified Delphi process was chosen to base our research on the current literature; however it could have limited the inclusion of additional important attributes. The fact that there were no attributes added to the list after the first round, in which participants were specifically asked if there were other attributes to include, suggests that this method may not have significantly impacted our results.

## Conclusion

Through a modified Delphi process of experienced medical educators, we identified and defined 10 foundational non-clinical attributes necessary for graduating medical students to be best prepared for residency training. These attributes can be used to guide curriculum development in the fourth year of medical school, or even earlier.

## Abbreviations

ACGME: Accreditation Council for Graduate Medical Education
EPA: Entrustable Professional Activity
PCRS: Physician Competency Reference Set
